# Survival of in-hospital cardiac arrest in men and women in a large Swedish cohort

**DOI:** 10.1186/s13049-018-0576-0

**Published:** 2018-12-19

**Authors:** Angelika Qvick, Manar Radif, Caroline Brever, Jenny Olsson Myrvik, Karin Schenk Gustafsson, Therese Djärv

**Affiliations:** 1 Function of Emergency Medicine, Karolinska University Hospital, Stockholm, Sweden; 2 Department of Medicine Solna, Cardiac Unit, Center for Gender Medicine, Karolinska Institutet and University Hospital, Solna, Sweden; 3 Department of Medicine Solna, Karolinska Institutet, Stockholm, Sweden

**Keywords:** Gender, Sex, 30-day survival, IHCA

## Abstract

**Background:**

Cardiac arrest is more common in men than women and a few studies have shown inferior 30-day survival for men than for women. The difference might relate to patient characteristics, intra arrest factors or post arrest care.

**Aim:**

To assess differences in 30-day survival between men and women after an in-hospital cardiac arrest (IHCA).

**Material and methods:**

All patients ≥18 years suffering an IHCA at Karolinska University Hospital between 2007 and 2017 were included. Data regarding the IHCA, patient characteristics, Charlson co-morbidity index (CCI) and 30-day survival were obtained from electronic patient records. Differences in survival between men and women were assessed with adjusted logistic regression models and presented as Odds Ratios with 95% Confidence Intervals (OR, 95% CI). Adjustments included age, CCI, place of cardiac arrest, first rhythm, ECG-surveillance and witnessed or not.

**Results:**

In all, 1639 patients suffered an IHCA, of whom 650 (40%) were women and 193 (30%) of them survived to 30 days compared to 28% of the men. No differences were found in the studied patient characteristics, intra arrest factors or post-ROSC treatments. Men had similar survival as women (crude OR 0.93 95% CI 0.74–1.15 and adjusted OR 0.77 95% CI 0.58–1.03 respectively).

**Conclusion:**

This cohort study illuminates an almost equal distribution in characteristics and treatment as well as outcome, 30-day survival after IHCA between men and women. However, our study confirms previous findings of disadvantageous prerequisites among women, but also indicates that preceeding vital signs differ which might indicate residual confounding.

## Introduction

The impact of sex on survival after cardiac arrest remains in the current situation somewhat unclear with conflicting results. Previous studies show that men are overrepresented in the cardiac arrest population, constituting approximately 60% of all cases but have about a 10% lower chance of 30-day survival than women [1–3]. This decreased survival for men has been shown despite unfavourable prehospital factors among out-of-hospital cardiac arrests (OHCA) such as higher age, fewer witnessed arrests and less likely to receive bystander-CPR for women [3]. Also, women with in-hospital cardiac arrest (IHCA) have both higher age and prevalence of non-shockable first rhythm [2]. The phenomenon that these studies demonstrate has been described as the ‘gender paradox’, where women survive cardiac arrests to a greater extent despite disadvantageous prerequisites [4]. One explanatory factor proposed for this ‘gender paradox’, have been that women are older when getting coronary artery disease, the reason for this is still unsolved, a so-called cardiovascular protective ‘oestrogen effect’ has been proposed [3, 5]. Another explanatory factor was presented in an American study from 2014, where female gender was associated with longer CPR-duration in patients with an IHCA not achieving ROSC [6].

Still, there are conflicting results and sex has not been shown as a prognostic factor for survival after an IHCA [7]. In summary, women appear to have better outcome than men regarding cardiac arrests and the causal factor of this phenomenon is yet unknown. Therefore, we conducted a cohort study with the aim of assessing differences in patient characteristics, intra arrest factors as well as post-ROSC treatments between men and women as well as the association with gender and 30-day survival after an IHCA.

## Method

### Study design and settings

This cohort study took place at Karolinska University Hospital (Karolinska) between 1st January 2007 and 31st December 2017. Karolinska, in Stockholm, home to approximately 2,000,000 people, is one of five large Stockholm hospitals. Karolinska has two sites 30 km apart, Solna and Huddinge. The Solna site hosts about 750 beds and is a level one trauma unit, has neuro and thoracic surgery units and provides 24/7 angiography for ST-elevation myocardial infarctions. The Huddinge site also hosts about 750 beds - and relatively fewer intensive care unit (ICU) beds. Karolinska as a whole has about 108,000 admissions yearly and 1.8 million patient visits. Karolinska follows Swedish guidelines for resuscitation based on European Resuscitation Councils guidelines.

### Ethics

This study used a database and registry: all survivors are informed about their participation at 6 months after their IHCA and can at any time afterwards withdraw their inclusion in the Swedish Registry for Cardio-Pulmonary Resuscitation (SRCR). Since the start of the registry in 1990 only a handful of patients have withdrawn their participation. Non-survivors are included without informed consent. The Regional Ethical Review Board in Stockholm, Sweden approved the study, Dnr 2013/1959–31/4.

### Participants

All cases of IHCA among adults, i.e. patients aged at least 18 years, occurring between January 1st 2007 and December 31th 2017 were eligible for inclusion in the study and were identified through the hospital’s cardiac arrest report sheet which is developed for collecting variables for participation in the SRCR. The SRCR [8, 9] collects data according to Utstein template [10], and the SRCRs definition of IHCA was used in the current study, i.e. a hospitalised patient who is unresponsive with apnoea (or agonal, gasping respiration) where CPR and/or defibrillation have been initiated. No patients or location of the IHCA were excluded. In the case of multiple IHCAs, only the first event was included.

### Data collection and categorisation

Patients were identified through the hospital’s cardiac arrest report sheet, where data on the following variables were collected: sex, age (collected in years, categorised in 10-year intervals starting at 18–50, 51–60 and further on to > 81 years), location of IHCA (general ward, intermediate care unit, intensive care unit (ICU), angio lab/operation theatre or other area including emergency department and radiology department), and first documented heart rhythm (VT/VF or PEA/asystole). Thereafter by entering the hospital’s electronic patient record (Take Care version 14.2.9) information on co-morbidities was gathered based on ICD-10 codes available at least at admission to the hospital and assessed according to the Charlson co-morbidity index (CCI) [11, 12] and categorised into “*None”* if CCI was 0 points, “*Low burden of co-morbidities*” if the CCI was 1–2 points, “*Moderate burden of co-morbidities*” if the CCI was 3–5 points or “*High burden of co-morbidities*” if the CCI was at least 6 points.

For a subset of the population, i.e. IHCA occurring 2015–2017 information on two additional variables was available in the medical file and therefore collected, i.e. information on National Early Warning Score (NEWS) [13] as well as information of a decision to withhold or withdraw life-sustainable treatment post- ROSC. Information regarding NEWS was collected as close as possible and maximum 12 h before the IHCA and categorized into low (0–4 points), medium (5–6 points or 3points in one single parameter) or high (at least 7 points) if information was available on all 7 included parameters.

Information on the outcome, i.e. 30-day survival (yes or no) was retrieved through the electronic patient record which is linked to the Swedish total population registry and automatically updated within a maximum delay of three days, which enables a complete follow-up [14].

### Statistical analyses

Characteristics of women and men were compared using the two-sided Chi [2] test and a *p*-value of ≤0.05 was interpreted as statistically significant. Missing data were not excluded or imputed, yet instead kept as an own category per variable. In order to assess the association between gender and 30-day survival, a logistic regression model was used and results were expressed as odds ratios (OR) with 95% confidence intervals (95% CI) with adjustment a priori decided and for known confounders: 1) age 2) CCI 3) place of IHCA 4) witnessed 5) ECG-surveillance 6) first documented heart rhythm. All analyses were performed with the statistical package STATA 10.2 for Windows (STATA Corp, College Station, TX).

## Results

### Patient characteristics

In all, 1639 patients suffered an IHCA at Karolinska during 2007–2017 and the overall 30-day survival ratio was 29%. The portion of women was 40% (*n* = 650) and their 30-days survival was 30% compared to 28% for the 989 (60%) men. There was an almost equal distribution age among women and men except for that more men were aged 61–70 years (27% versus 20%, respectively) and more women aged at least 81 years (30 and 21%, respectively, *p*-value< 0.01, Table 1). No statistically significant differences were found regarding Charlson co-morbidity Index.

**Table 1:** Characteristics of 1639 patients suffering an in-hospital cardiac arrest during 2007–2017 in Karolinska University Hospital

	Women Number (%) 650 (100)	Men Number (%) 989 (100)	*P*-value*
Age category			< 0.01
18–50	50 (8)	101 (10)	
51–60	84 (13)	117 (12)	
61–70	131 (20)	271 (27)	
71–80	192 (30)	288 (29)	
≥ 81	193 (30)	212 (21)	
Charlson co-morbidity index			0.64
None 0 points	222 (34)	346 (35)	
Low 1–2 points	226 (35)	331 (33)	
Moderate 3–5 points	152 (23)	220 (22)	
High 6–8 points	50 (8)	92 (9)	
National Early Warning Score 2015–2017**	240 (100)	207 (100)	0.10
Low 0-4points	97 (40)	66 (32)	
Medium 5–6 points or a single 3 point	46 (19)	50 (24)	
High, at least 7 points	44 (18)	31 (15)	
Missing	53 (22)	60 (29)	
Place of cardiac arrest			0.09
Patient ward	261 (40)	401 (41)	
High Dependency Unit	163 (25)	234 (24)	
Intensive care unit	57 (9)	105 (11)	
Operating/procedure rooms	11 (2)	19 (2)	
Angio cath lab	38 (6)	86 (9)	
Emergency Department	66 (10)	89 (9)	
Others incl. x-ray department	54 (8)	55 (6)	
ECG- surveillance			0.01
Yes	299 (46)	509 (51)	
No	343 (53)	453 (46)	
Missing	8 (1)	27 (3)	
Witnessed cardiac arrest			0.20
Yes	522 (80)	766 (72)	
No	119 (18)	206 (21)	
Missing	9 (1)	17 (2)	
First documented heart rhythm			< 0.01
VT/VF	105 (16)	241 (24)	
PEA/asystole	422 (65)	623 (63)	
Missing	123 (19)	125 (13)	
CRP duration (minutes)			
All, Median (IQR)	8 (2–18)	6 (2–16)	0.14
ROSC	5 (2–11)	5 (2–12)	
No ROSC	22 (14–35)	20 (12–30)	
ROS			0.09
Yes	350 (54)	490 (50)	
Decision to withdraw/withhold life-sustainable treatments among those having ROSC 2015–2017			
Yes	25 (17^**€**^)	22 (17^**€**^)	
Hypothermia/Target Temperature Management			
Yes	27 (8***)	66 (13***)	0.11

*P-values were assessed with Chi2-test regarding differences between women and men

** NEWS was only available during the years 2015–2017

€ Information only available for IHCA during the years 2015–2017, i.e. out of 240 women and 207 men having an IHCA, 150 (63%) and 124 (60%) had ROSC, respectively. Percentage 25/150 and 22/124 respectively

*** Percentage = number with treatment/number with ROSC

### Cardiac arrest factors

Regarding preceding warning signs, no statistically significant difference was found regarding NEWS category among men and women, however more men had higher categories and missing information (Table 1). More men than women suffered their IHCA under ECG-surveillance (51% versus 46%, *p*-value < 0.01, Table 1). Regarding the first documented rhythm, the portion of non-shockable were equal but more men had shockable rhythms and more women had missing information (p-value < 0.01, Table 1). Duration of CPR attempts was equal between men and women both among those having ROSC and those not having ROSC (Table 1).

Likewise, no statistically significant differences were found regarding place of IHCA, witnessed or not or portion having ROSC.

### Post- ROSC factors

No statistically significant difference was found on any post-ROSC variable, i.e. a decision to withdra*w*/withhold life-sustainable treatments among those having ROSC or treatment with hypothermia (2007–2015) or target temperature management (2016–2017) (Table 1).

### Association between sex and 30-day survival

Men had an almost equal OR for 30-day survival compared to women (crude OR 0.93 95% CI 0.74–1.15) that remained after adjustment for known confounders (adjusted OR 0.77 95% CI 0.58–1.03, Table 2).

**Table 2:** Association between sex and 30-day survival after in-hospital cardiac arrest at Karolinska University Hospital in 2007–2017

Sex	Number of patients	30 days survival Number (%)	Crude OR (95% CI)	Adjusted OR (95% CI)*
Women	650	193 (30)	1.00 (Reference)	1.00 (Reference)
Men	989	278 (28)	0.93 (0.74–1.15)	0.77 (0.58–1.03)

*Adjusted for age, Charlson Comorbidity Index [23], location of cardiac arrest, first documented heart rhythm, ECGsurveillance and witnessed or not

## Discussion

This cohort study over 10 years illuminates an almost equal distribution of patient characteristics and cardiac arrest factors and possibly therefore an equal 30-day survival after an IHCA for men and women.

Still this study confirms previous findings of disadvantageous prerequisites such as higher age in women and more often shockable rhythm in men, but also indicates that men might have more severely deranged vital signs or not having their vital signs checked equally often as women which deserves future attention and might indicate residual confounding.

The results on age and shockable rhythm are in line with previous studies [2, 15], but no previously published study has investigated sex differences in CCI, NEWS and decision to withhold/withdraw treatment post-ROSC among a cardiac arrest population, and therefore comparisons to other studies are not possible. A factor affecting both the incidence to have an IHCA and the outcome of such is the admission to intensive care. Previous studies have shown that women are less likely to receive early invasive strategies such as PCI [16–18] as well as being admitted to ICU as well as received less advanced treatment while at the ICU, discussed to be a consequence of more assessments by Rapid Response Teams in men and more decisions to withhold/withdraw treatment in women [19]. However this could not be shown within our study. Interestingly, the same research group has shown that there is no difference in willingness to admit men or women to ICU but female physicians tended to be more willing to admit patients, regardless of the patient being a man or a woman, than their male counterparts [20]. Also, women tend to be placed in ordinary internal medicine unites instead of CCU’s [21, 22].

Regarding the previously shown increased early survival, i.e. ROSC or admitted alive to hospital for women [3, 4], we found no difference in either portion having ROSC or CPR durations between men and women.

The disadvantageous prerequisites for women compared to men in studies regarding OHCA were identified as higher age, more likely to present with an OHCA in a non-public space, less likely to have a witnessed OHCA and a lower frequency of initial shockable rhythm, [2]. Solely age and initial rhythm are directly translatable in a hospital setting, which causes issues when comparing with this study.

Limitations of the study include residual confounding in variables not captured within this study such as PCI and etiology of the cardiac arrest which is important since cardiac etiologies are more common in shockable rhythms than non-shockable one. Further, factors involved in and affecting daily care not even known to clinicians and researchers are a major limitation. Further, we lack information about intake of estrogen among women, however Swedish guidelines do not recommend estrogen in women of the ages having IHCA [21].

Strengths include the inclusion of two separate different hospital-sites over ten years. Further, collection of data was based on a strict a priori determined protocol and another strength is the complete information about the outcome, i.e. 30-day survival or not due to Swedish Personal Identification Numbers and registries [14].

In conclusion, this is the first study following patients with IHCA comparing men and women over 10 years. We found equal distribution of patient characteristics and cardiac arrest factors and possibly therefore an equal 30-day survival after an IHCA for men and women.

However, our study confirms previous findings of disadvantageous prerequisites such as higher age in women and more often shockable rhythm in men, but also indicates that men might have more severely deranged vital signs or not having their vital signs checked equally often as women which deserves future attention and might indicate residual confounding.

## Abbreviations

CCI: Charlson co-morbidity index
CPR: Cardio-pulmonary resuscitation
ICU: Intensive care unit
IHCA: In-hospital cardiac arrest
Karolinska: Karolinska University Hospital
NEWS: National Early Warning Score
OHCA: Out-of-hospital cardiac arrest
OR, 95% CI: Odds Ratios with 95% Confidence Intervals
PCI: Percutan coronary intervention
PEA: Pulseless electric activity
ROSC: Return of spontaneous circulation
SRCR: Swedish Registry for Cardio-Pulmonary Resuscitation
VF: Ventricle fibrillation
VT: Ventricle tachycardia
